# Histone Demethylation Maintains *Prdm14* and *Tsix* Expression and Represses *Xist* in Embryonic Stem Cells

**DOI:** 10.1371/journal.pone.0125626

**Published:** 2015-05-20

**Authors:** Yasunao F. Kamikawa, Mary E. Donohoe

**Affiliations:** 1 Burke Medical Research Institute, White Plains, New York, United States of America; 2 Department of Neuroscience Brain Mind Research Institute, Weill Cornell Medical College, New York, New York, United States of America; 3 Department of Cell & Development, Weill Cornell Medical College, New York, New York, United States of America; National University of Singapore, SINGAPORE

## Abstract

Epigenetic reprogramming is exemplified by the remarkable changes observed in cellular differentiation and X-chromosome inactivation (XCI) in mammalian female cells. Histone 3 lysine 27 trimethylation (H3K27me3) is a modification that suppresses gene expression in multiple contexts including embryonic stem cells (ESCs) and decorates the entire inactive X-chromosome. The conversion of female somatic cells to induced pluripotency is accompanied by X-chromosome reactivation (XCR) and H3K27me3 erasure. Here, we show that the H3K27-specific demethylase Utx regulates the expression of the master regulators for XCI and XCR: *Prdm14*, *Tsix*, and *Xist*. Female ESC transcriptome analysis using a small molecule inhibitor for H3K27 demethylases, GSK-J4, identifies novel targets of H3K27 demethylation. Consistent with a recent report that GSK-J4 can inhibit other histone demethylase, we found that elevated H3K4me3 levels are associated with increased gene expression including *Xist*. Our data suggest multiple regulatory mechanisms for XCI via histone demethylation.

## Introduction

Post-translational modifications (PTMs) of histones are major players in epigenetic regulation and are required for multiple genomic functions such as DNA replication and gene transcription [1,2]. The histone 3 lysine 27 tri-methylation (H3K27me3) mark is enriched at a subset of genomic loci that are temporally repressed and poised for reactivation upon proper stimuli [3]. A remarkable feature of H3K27me3 is that it is enriched along the entire inactive X-chromosome (Xi) in mammalian female somatic cells [4,5]. During cellular differentiation, one of two female X-chromosomes is epigenetically silenced to balance the X-linked gene dosage with XY males in a process called X-chromosome inactivation (XCI). XCI is governed by two long noncoding RNAs (lncRNAs): *Xist* the silencer, and *Tsix* the antisense counterpart of *Xist*. *Xist* expression becomes allele-specific from the future Xi and is robustly expressed during the XCI process. In contrast, *Tsix* is highly expressed in the pluripotent state and represses *Xist* expression [6]. Consistent with its expression pattern, *Tsix* is regulated by several pluripotent factors such as Oct4, Sox2 and Rex1 [7,8]. Upon cellular differentiation, *Tsix* expression is progressively reduced allowing *Xist* elevation. During the reprogramming of female somatic cells back to an induced pluripotent state (induced pluripotent stem cells (iPSCs)), the entire X-chromosome is reactivated (X-chromosome reactivation (XCR)), *Tsix* expression increases, *Xist* expression is extinguished, and the H3K27me3 PTM is erased from the inactive X. The mechanism for this erasure in XCR is not known although recent studies reveal several pluripotent factors such as Prdm14, Klf2, and *Tsix* trigger XCR [9,10]. These findings prompted us to ask whether H3K27 demethylases play a role in regulating pluripotency and the XCI/XCR cycle.

The ubiquitously transcribed tetratoricopeptide on X (Utx) and Jumonji-C domain-containing protein 3 (Jmjd3), encoded by *Kdm6a* and *Kdm6b*, respectively, have been identified as H3K27me2/me3-specific demethylases [11,12]. Previous studies have shown multiple functions of these proteins in normal development and cellular reprogramming [12,13]. Intriguingly, a genome-wide screening revealed that Utx is required for the reprogramming of somatic cells to iPSCs and germ cells [14]. Here, we elucidate the function of H3K27me3 demethylation for the expression of pluripotent genes and the suppression of XCI using female ESCs. We find that a small molecule GSK-J4, originally established as a selective inhibitor for H3K27 demethylases, can activate gene expression by inhibiting other JmjC domain demethylases such as H3K4me3, consistent with a recent report [15]. Our results show that histone demethylases play a dynamic role in XCI.

## Results

### Inhibition of demethylases by GSK-J4 treatment results in reduced expression of pluripotent genes

**Fig 1:**
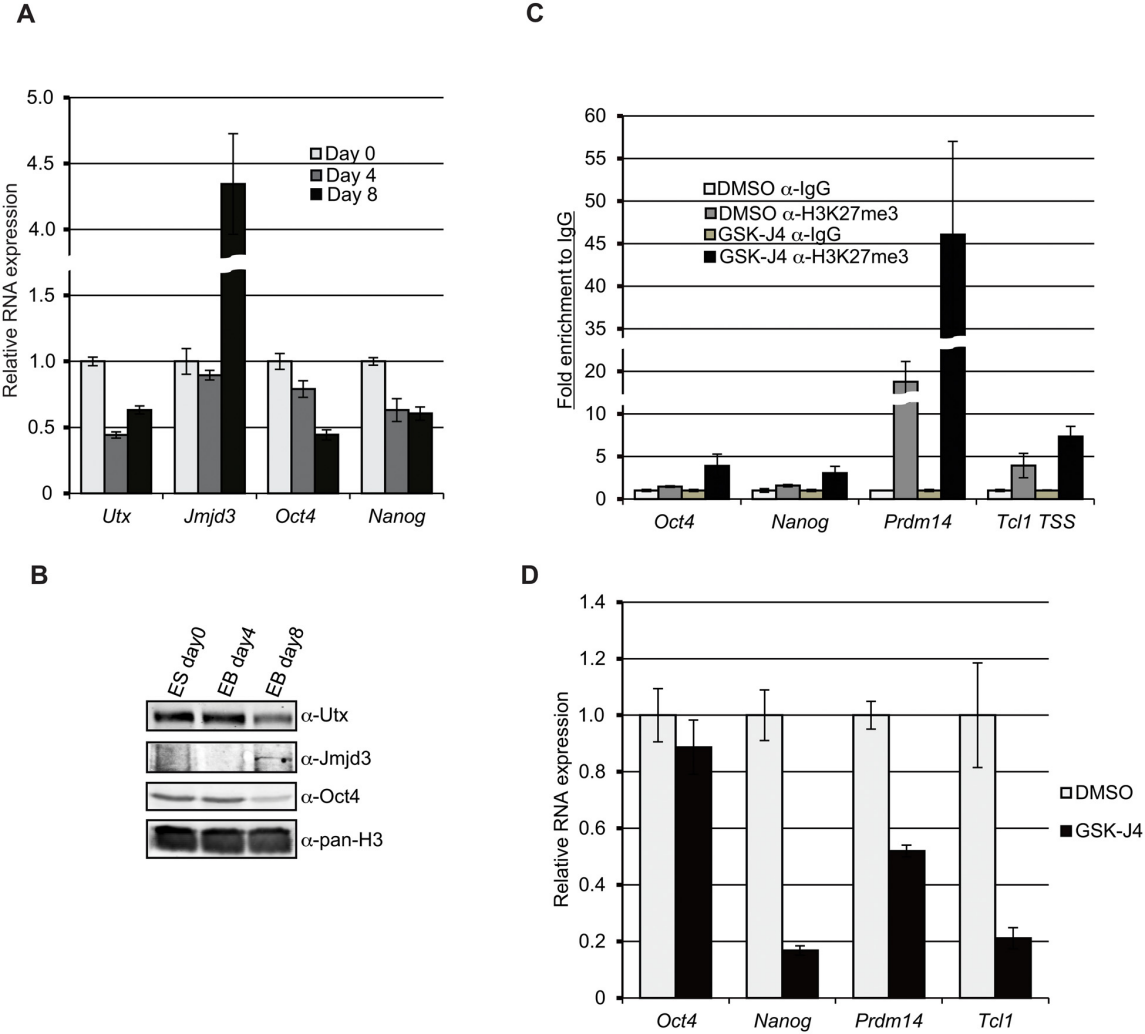
GSK-J4 treatment results in reduction of *Nanog*, *Prdm14*, and *Tcl1* expression. (A) RNA expression of *Utx* and *Jmjd3* during the differentiation of female ESCs. ESCs were differentiated by removal of LIF and forming embryoid bodies (EBs). RNA was extracted from undifferentiated ESCs (day 0 (d0)), day 4 (d4) and day 8 (d8) of differentiation. Relative RNA expression was determined with RT-qPCR. The graph represents mean values of three independent experiments. Error bars show one standard deviation from the mean. (B) Western blot analysis of Utx, Jmjd3, and Oct4 during differentiation. Histone H3 was used a protein loading control. (C) LF2 ESCs were treated with 10 μM GSK-J4 for 24 hr and subjected to quantitative chromatin immunoprecipitation (qChIP) using anti-H3K27me3 antibodies and primer sets for the transcriptional start sites (TSSs) of the indicated genes. The graph represents mean values of fold enrichment relative to IgG control from three independent experiments. Error bars represent on standard deviation from the mean. (D) Alteration of *Oct4*, *Nanog*, *Prdm14*, and *Tcl1* expression following GSK-J4 treatment. LF2 ESCs were treated by 10 μM GSK-J4 for 24 hr and RNA levels were measured by RT-qPCR. The graph represents the mean values of three independent experiments. Error bars show one standard deviation from the mean.

First, we measured the temporal expression levels of Utx and Jmjd3 during the cellular differentiation of female mouse ESCs by forming embryoid bodies (EBs) and the concomitant removal of leukemia inhibitory factor (LIF). The cells were harvested at the designated differentiation days. Consistent with previous reports using male ESCs [16, 17], the mRNA levels of *Utx* decline while *Jmjd3* increases during the differentiation of female ESCs (Fig 1A). Similar to the mRNA data, the Utx protein level is reduced in day 8 female EBs (Fig 1B). In contrast, we can only detect Jmjd3 protein at day 8 of differentiation (Fig 1B). We could not observe Jmjd3 protein following inhibition of the proteasome with MG-132 treatment suggesting that the lack of Jmjd3 protein is not due to its degradation by the proteasome (S1 Fig). Due to the low expression of Jmjd3 protein in undifferentiated ESCs, we focus our studies here on Utx.

To elucidate the roles of H3K27 demethylation on the expression of pluripotent genes, we treated female undifferentiated ESCs with GSK-J4, a small molecule inhibitor specific for Utx and Jmjd3 H3K27 demethylase catalytic activity [18]. Inhibition of H3K27me3 demethylation following GSK-J4 treatment was confirmed by quantitative chromatin immunoprecipitation (qChIP) using anti-H3K27me3 antibodies at the transcriptional start sites (TSSs) of the pluripotent genes *Oct4*, *Nanog*, *Prdm14*, and *Tcl1* (Fig 1C). Indeed, we observe an increase of H3K27me3 at these loci following GSK-J4 exposure, in particular at the *Prdm14* TSS, which shows the highest signal of H3K27me3 in both the control and the GSK-J4 treated ESCs. The expression levels of these genes were measured by reverse-transcription, quantitative PCR (RT-qPCR). *Nanog*, *Prdm14*, and *Tcl1* show reduced expression with GSK-J4 treatment although the expression of *Oct4* is slightly decreased (Fig 1D). Two independent male and female ESCs treated with GSK-J4 confirms the altered gene expression (S2 Fig). Taken together, these results suggest that the H3K27 demethylase activity is necessary for the expression of *Nanog*, *Prdm14*, and *Tcl1*.

### GSK-J4 diminishes *Tsix* and induces *Xist* expression

**Fig 2:**
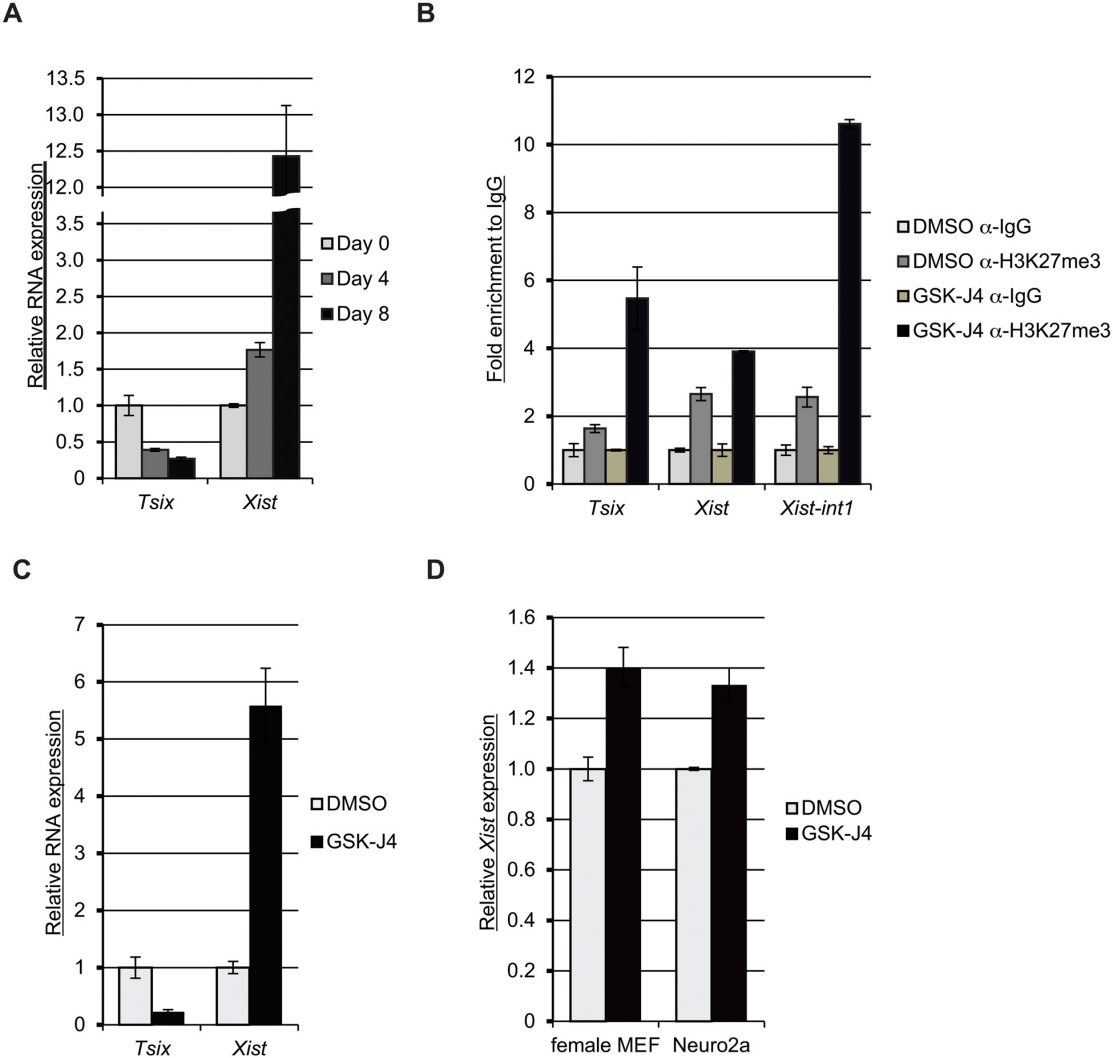
GSK-J4 demethylase inhibitor extinguishes *Tsix* and induces Xist expression. (A) RNA expression levels of *Tsix* and *Xist* during the differentiation of female ESCs. RNA was extracted from indicated stages of cells and subjected to RT-qPCR. The graph is representative of mean values of three independent experiments. Error bars represent one standard deviation from the mean. (B) Female ESCs were treated with 10 μM GSK-J4 and subjected to qChIP at the TSSs of *Tsix* and *Xist*, as well as *Xist* intron 1 (*Xist*-int1) using anti-H3K27me3 antibodies. The graph represents mean values of fold enrichment to IgG from three independent experiments. Error bars show one standard deviation from the mean. (C) Alteration of *Tsix* and *Xist* expression after GSK-J4 treatment. ESCs were treated with 10 μM GSK-J4 for 24 hr and subjected to RT-qPCR. Relative RNA expression is shown as mean values of three independent experiments. (D) Female MEFs and Neuro2a cells were treated with 10 μM GSK-J4 for 24 hr and the relative RNA expression of *Xist* was determined by RT-qPCR. The mean values of three independent experiments are shown. Error bars represent one standard deviation from the mean.

XCI in the mouse embryo can be faithfully recapitulated *ex vivo* by inducing the differentiation of female ESCs. Both female X-chromosomes are active in undifferentiated ESCs. During cellular differentiation, *Tsix* expression extinguishes and *Xist* is robustly upregulated reflecting the gradual silencing of the entire inactive X-chromosome (Fig 2A). Following GSK-J4 exposure, the level of H3K27me3 is increased at the TSSs of *Tsix* and *Xist*. We also observe enhanced H3K27me3 at *Xist* intron 1 (*Xist-int1*) (Fig 2B), where several pluripotent factors (such as Oct4, Nanog, and Sox2) bind [7,8]. We then tested the effect of inhibition of H3K27me3 demethylation on the expression of *Tsix* and *Xist* in undifferentiated female ESCs. GSK-J4 treatment reduces *Tsix* and increases the expression of *Xist* (Fig 2C).

These results suggest that H3K27me3 demethylation is indispensable for the maintenance of *Tsix* expression and for preventing ectopic *Xist* expression. Because *Tsix* is a repressor for *Xist*, we asked whether ectopic activation of *Xist* is dependent on *Tsix* repression. To answer this question, we treated female primary mouse embryonic fibroblasts (MEFs) [19], which do not express *Tsix*, with GSK-J4 and measured the *Xist* levels. We observed approximately 40% increase of *Xist* expression after GSK-J4 treatment (Fig 2D). The induction of *Xist* expression in female MEFs is more robust than that of ESCs with the basal MEF *Xist* levels at least 100 fold higher expression than undifferentiated ESCs [20], suggesting that the induction of *Xist* by GSK-J4 is at least partially, independent of *Tsix* repression. In contrast to the male undifferentiated ESCs, GSK-J4 treatment did not affect *Xist* expression in male MEFs (S3 Fig). We confirmed this increased expression of *Xist* by GSK-J4 treatment in Neuro2a cells, a female mouse neuroblastoma cell line (Fig 2D). Our results suggest that H3K27 demethylation is required for *Tsix* expression and the repression of *Xist*.

### Utx occupies the transcriptional start sites of *Prdm14*, *Tsix*, and *Xist* intron 1 and regulates their expression

**Fig 3:**
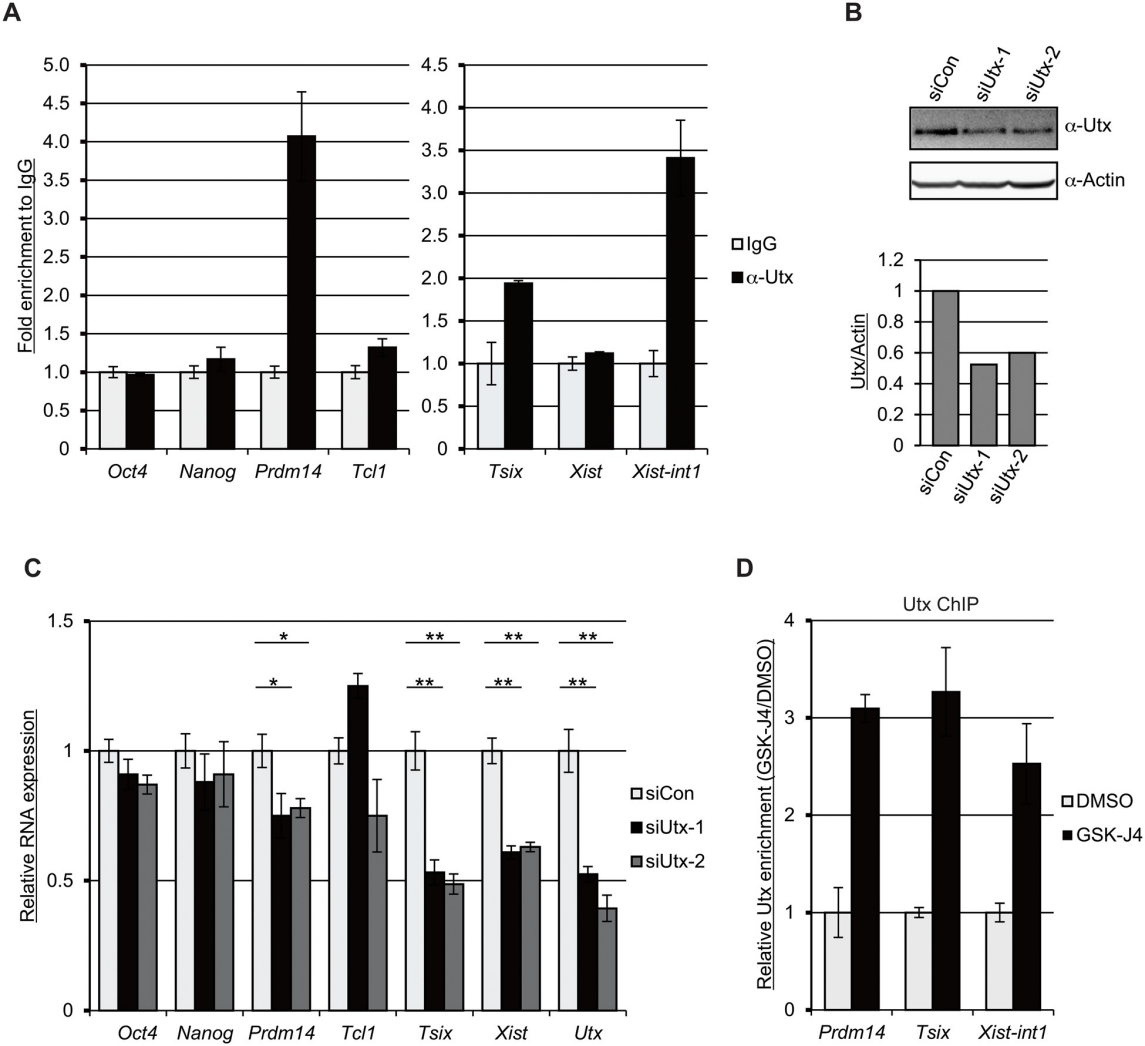
Utx binds to the transcriptional start sites (TSSs) of *Prdm14*, and *Tsix*, and *Xist intron 1* and regulates these genes in ESCs. (A) Female ESCs were subjected to qChIP using anti-Utx antibodies and the primer sets for the TSSs of *Oct4*, *Nanog*, *Prdm14*, *Tcl1*, *Tsix*, and *Xist*; as well as *Xist* intron 1 (*Xist*-int1). The graphs represent the mean fold values of enrichment relative to IgG control from three independent experiments. Error bars show one standard deviation from the mean. (B) Female ESCs were transfected with a control siRNA (siCon) and two different siRNAs for Utx (siUtx-1 and siUtx-2). The transfectants were subjected to western blot with anti-Utx antibodies 72 hr post transfection. Actin is used as a protein loading control. The graph represents the fold change of Utx and Actin proteins. (C) The relative RNA expression was measured by RT-qPCR in the Utx depleted cells. The graph represents the mean values of three independent experiments. Error bars represent one standard deviation from the mean. Student’s *t*-test (two tailed unpaired) was used for statistical analysis. *p<0.05; **p<0.01. (D) Female ESCs were treated with 10 μM of GSK-J4 for 24 hr and then subjected to qChIP using anti-Utx antibodies.

Using qChIP we tested whether the Utx demethylase occupies the TSSs of *Oct4*, *Nanog*, *Prdm14*, *Tcl1*, *Tsix*, *Xist*, and *Xist intron 1* in undifferentiated female ESCs. We found that Utx is enriched at the TSSs *Prdm14*, *Tsix*, and *Xist intron 1*, but not at the TSSs of *Oct4*, *Nanog*, *Tcl1*, *or Xist* (Fig 3A). To confirm its functional importance in the expression of these genes, we depleted *Utx* using small interfering RNAs (siRNAs) targeting two different regions of the *Utx* gene. Western blot shows that Utx protein is reduced (approximately 50%) following knockdown (Fig 3B). Consistent with the GSK-J4 treatment, RT-qPCR reveals a reduced expression of *Prdm14* and *Tsix* in the Utx knockdown cells (Fig 3C). In addition, we observe a decrease in *Xist* expression following Utx reduction, indicating the difference of inhibiting demethylase activity versus the depletion of Utx (Fig 3C). Indeed, previous studies show that Utx can activate its targets expression in a demethylase-independent manner [21,22]. Both Utx and its family member, Uty, demonstrate histone demethylase-independent functions in mouse embryonic development. Interestingly, we found that Utx enriches at target loci upon GSK-J4 treatment (Fig 3D) even though the total Utx protein level is not changed (S4A Fig), suggesting that inhibition of H3K27 demethylation activates the demethylase-independent function of Utx Taken together, our data indicate that Utx directly regulates the expression of *Prdm14* and *Tsix* in a demethylase-dependent manner, and suggest that Utx controls *Xist* via demethylase-independent mechanisms.

### Ascorbic acid enhances the demethylase activity of Utx and induces its target genes

**Fig 4:**
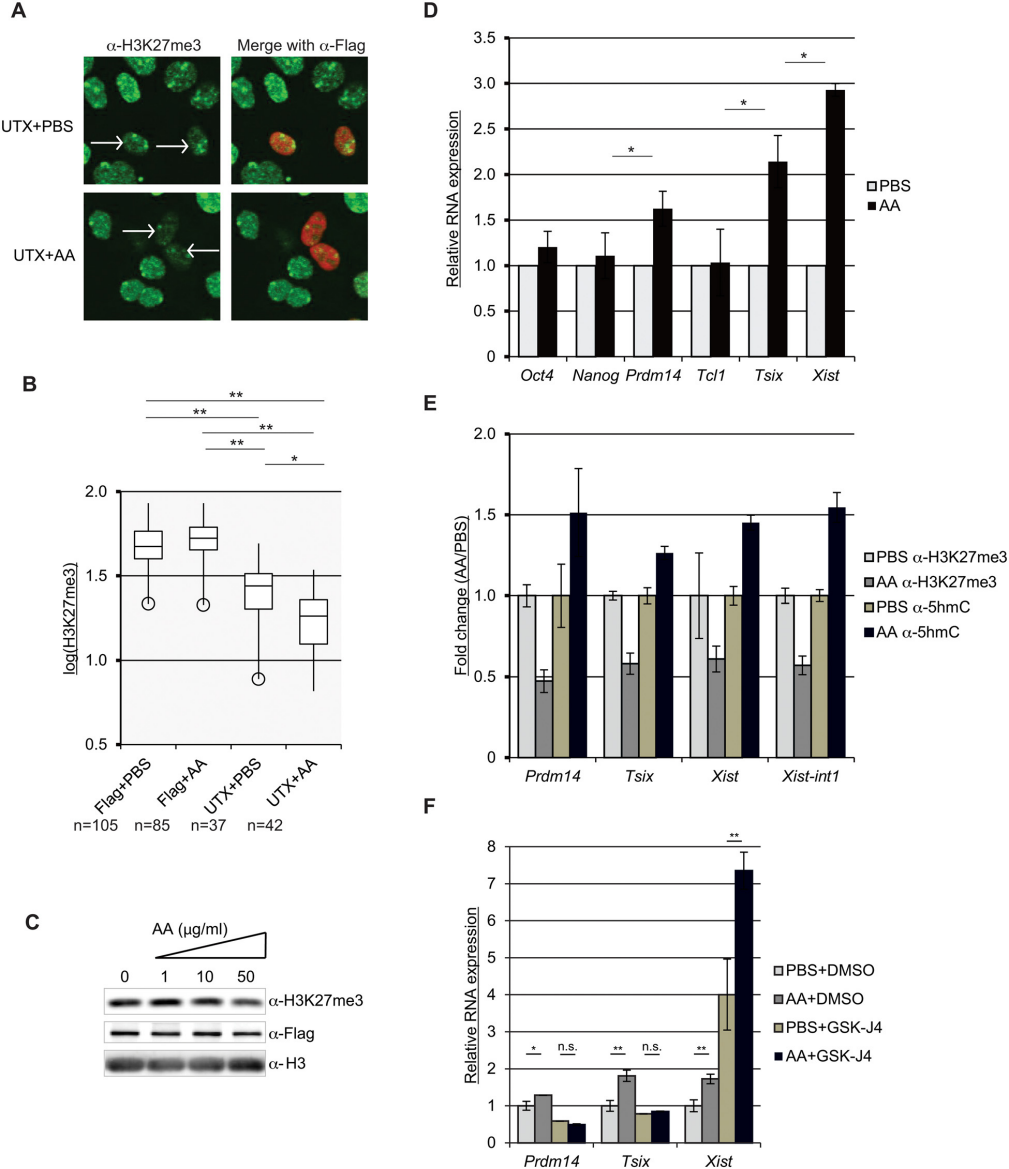
Ascorbic acid enhances demethylation of H3K27me3 and induces *Prdm14*, *Tsix*, and *Xist*. (A) The catalytic domain of Flag-tagged UTX protein was overexpressed in HEK cells, in the presence or absence of ascorbic acid (AA). The transfectants were subjected in immunostaining using anti-H3K27me3 and anti-Flag antibodies. (B) The signal intensities of H3K27me3 from individual cells are shown as box plots in log scale. The numbers of the counted cells are indicated as “n”. The Tukey-Kramer method was used for statistical analysis. *p<0.05; **p<0.01. (C) *In vitro* demethylase assay was performed using lysates from Flag-Utx overexpressing cells with or without AA. Western blot was performed after the *in vitro* demethylase assay using anti-H3K27me3, anti-Flag, and anti-H3 antibodies. (D) AA induces *Prdm14*, *Tsix*, and *Xist* expression in ESCs. Female ESCs were cultured with 50 μg/ml of AA for 24 hr and the relative RNA expression was determined by RT-qPCR. The graphs show the mean values of three independent experiments. Error bars represent one standard deviation from the mean. Student’s *t*-test (two tailed paired) was used for statistical analysis. * p<0.05. (E) The H3K27me3 levels are decreased while the 5hmC levels are elevated following AA exposure. Female ESCs were treated with 50 μg/ml of AA for 24 hr and the relative enrichment of H3K27me3 and 5hmC were determined by qChIP. Fold changes relative to the control are shown as the mean values of three independent experiments. Error bars show one standard deviation from the mean. (F) GSK-J4 diminishes AA-induced up-regulation of *Prdm14* and *Tsix*, but enhances that of *Xist* expression. Female ESCs were treated with GSK-J4 in the absence or presence of AA and subjected to RT-qPCR. The mean values of three independent experiments are presented using the AA fold induction. Student’s *t*-test (two tailed unpaired) was used for statistical analysis. * p<0.05; **p<0.01; n.s. = not significant (p>0.05). Error bars represent one standard deviation from the mean.

L-ascorbic acid (AA)/Vitamin C is a potential activator of α-ketoglutarate-dependent oxygenases [23,24]. Although previous studies reveal that the demethylation of 5-methyl cytosine (5mC), histone 3 lysine 9 (H3K9), and histone 3 lysine 36 (H3K36) enhance after AA exposure [23,22,25], it is unknown whether AA regulates H3K27 demethylation. We therefore tested whether AA can facilitate the demethylase activity of Utx. To do this, we overexpressed HEK cells with a C-terminal catalytic domain of UTX protein fused with a nuclear localization signal sequence SV40NLS (UTX-C_SV40NLS_) [26] and evaluated the demethylase activity with or without AA by immunostaining with anti-H3K27me3 antibodies (Fig 4A). We found that AA treated cells show a statistically significant reduction in H3K27me3 signal intensity (Fig 4B). AA can also enhance demethylation of H3K27me3 using lysates from UTX-C_SV40NLS_-expressing cells (Fig 4C). These results indicate that AA enhances the demethylation of H3K27me3.

Next, we treated female ESCs with AA and evaluated the expression levels of the genes tested above. Consistently, the *Prdm14* and *Tsix* levels increase after AA (Fig 4D). In contrast, we found an increased expression of *Xist* in AA treated cells, suggesting an H3K27me3 demethylation-independent mechanism. Indeed, it has been reported that AA treatment induces the global demethylation of 5-methyl cytosine (5mC), converting 5mC to 5-hydroxy methyl cytosine (5hmC) in ESCs via a Ten eleven translocated (TET)-dependent manner [23]. We evaluated the levels of H3K27me3 and 5hmC at the TSSs of *Prdm14*, *Tsix*, and *Xist* as well as *Xist-int1* after AA treatment. The H3K27me3 levels are reduced and the 5hmC levels are increased at all the loci tested (Fig 4E), suggesting that AA activates the demethylation of both H3K27me3 and 5mC. We next asked whether the AA-induced gene expression is mediated by H3K27 demethylation, and therefore treated ESCs with AA plus GSK-J4. The induction of *Prdm14* and *Tsix* by AA is significantly diminished following GSK-J4 exposure (Fig 4F), indicating that AA activates these genes through the demethylation of H3K27me3. In contrast, *Xist* levels are elevated in the AA plus GSK-J4 treated cells as compared with AA or GSK-J4 alone (Fig 4F), presumably due to the synergistic effect of inhibition of H3K27me3 demethylation and the activation of 5mC demethylation by GSK-J4 and AA, respectively. The Utx total protein levels do not change after AA exposure (S4B Fig). Collectively our results show that AA activates the demethylation of H3K27me3 and enhances 5hmC levels resulting in an induction of *Prdm14*, *Tsix*, and *Xist* expression.

### Transcriptional signature of GSK-J4 treated female ESCs

**Fig 5:**
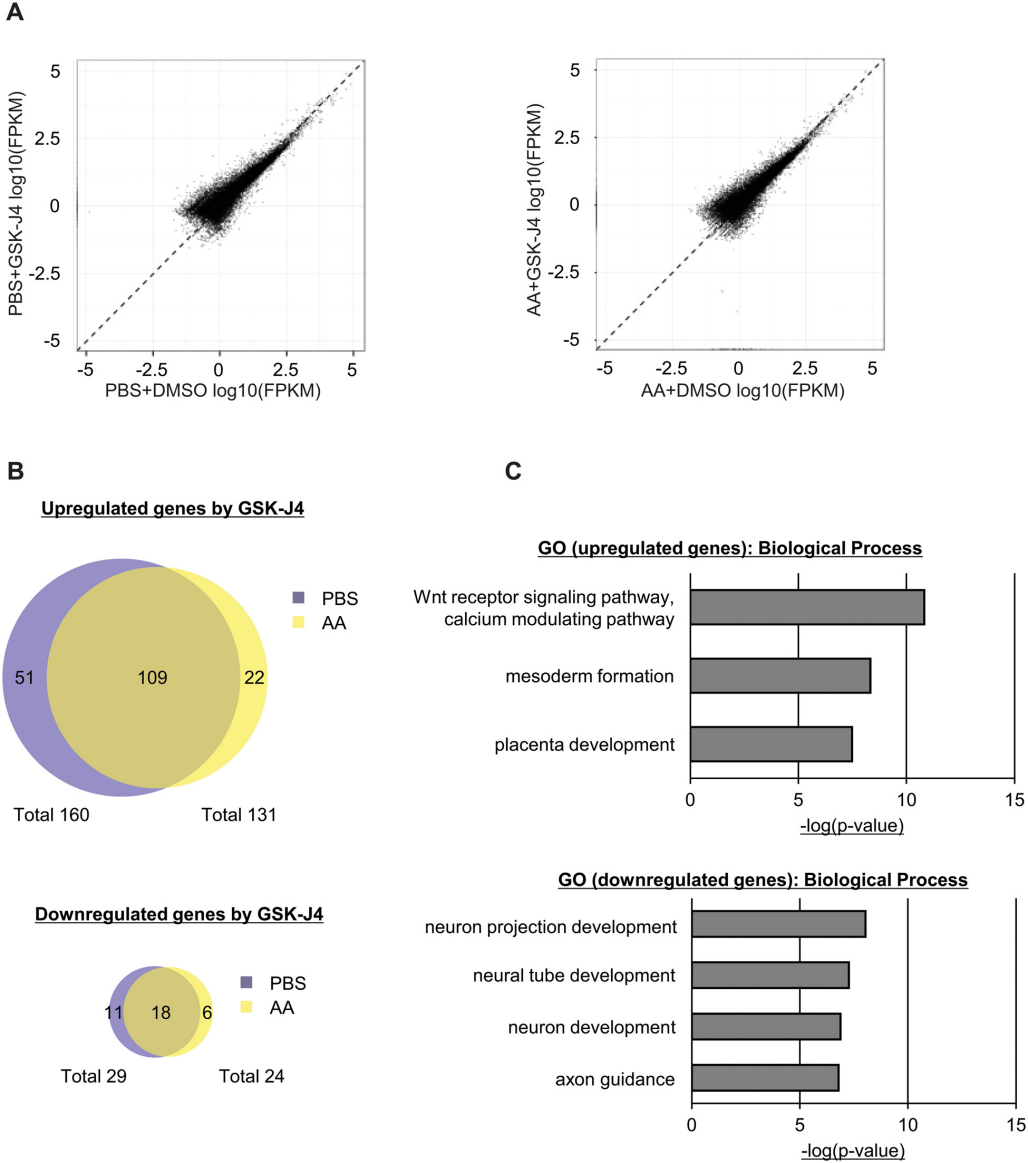
Transcriptome analysis of GSK-J4- and ascorbic acid-treated female ESCs. (A) Scatter plots between control and GSK-J4 treated cells in the absence of AA (left panel) and in the presence of AA (right panel) with FPKM. (B) Overlap between ESCs without AA and with AA treatment in up-regulated genes (top panel) and down-regulated genes (bottom panel) by GSK-J4 exposure. (C) Gene ontology analysis of differentially expressed genes. Top panel: up-regulated genes; Bottom panel: down-regulated genes.

**Fig 6:**
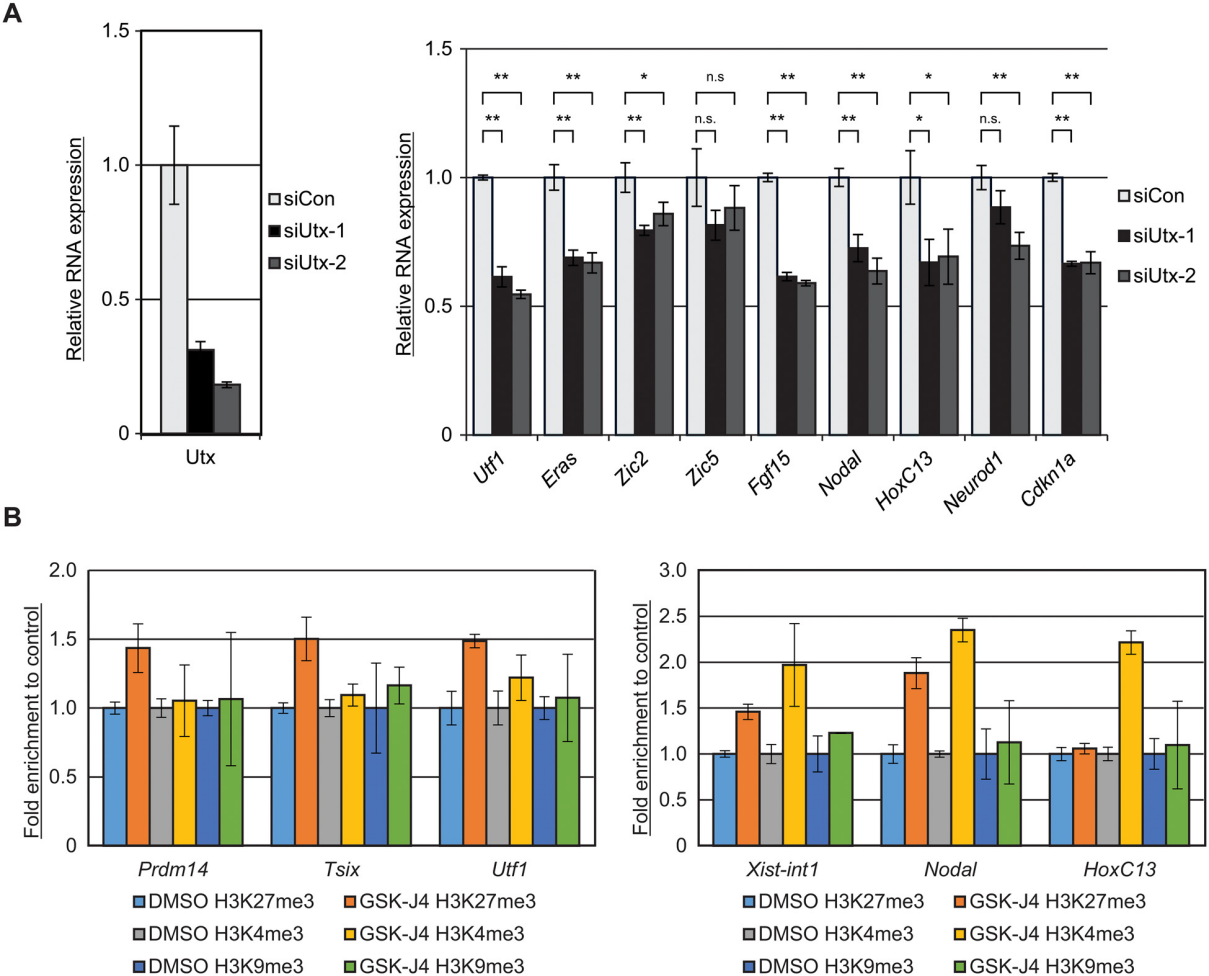
GSK-J4 induces gene expression by inhibiting H3K4me3 demethylation in ESCs. (A) Female ESCs were transfected with a control siRNA (siCon) and two different siRNAs targeting Utx (siUtx-1 and siUtx-2). RNA was extracted and subjected to RT-qPCR 72 hr after transfection. The graph represents the mean values of three independent experiments. The error bars represent one standard deviation from the mean. Student’s *t*-test (two tailed unpaired) was used for the statistical analysis. *p<0.05; **p<0.01; n.s. = not significant p>0.05. (B) GSK-J4 inhibits H3K4me3 demethylation and induces gene expression. Female ESCs were treated with 10 μM GSK-J4 for 24 hr and subjected to qChIP using anti-H3K27me3, anti-H3K4me3, and anti-H3K9me3 antibodies using primer sets spanning TSSs of *Prdm14*, *Tsix*, *Utf*, *Nodal*, and *HoxC13* and *Xist*-intron 1 (*Xist-int1*). The graph represents the mean values of fold enrichment relative to the control (DMSO) from three independent experiments. The error bars represent one standard deviation from the mean.

To further elucidate the biological relevance of H3K27 demethylation, we performed RNA-sequencing (RNA-Seq) on GSK-J4 treated female ESCs in the absence or presence of AA. The expression levels obtained from RNA-Seq were calculated as the FPKM (Fragments Per Kilobase of exon per Million mapped fragments (Fig 5A)). We confirmed the reduced expression of *Nanog*, *Prdm14*, and *Tcl* and induction of *Xist* (S5 Fig). We identified 189 statistically significant differentially expressed genes in the GSK-J4 cells without AA; whereas 155 differentially expressed genes are found in the GSK-J4 ESCs with AA exposure. Without AA, 160 genes are upregulated and 29 are repressed following GSK-J4 (Fig 5B, **top panel**, q-value<0.05). In the presence of AA, 131 of these differentially expressed genes are upregulated and 24 are decreased (Fig 5B, **bottom panel**, q-value<0.05). We analyzed the differentially expressed genes in the absence of AA using Gene Ontology (GO) analysis. Interestingly, the upregulated genes are enhanced for the Wnt signaling and calcium modulating pathways, mesoderm formation, and placental development (Fig 5C, **top panel**). In contrast, the downregulated genes are enriched for neuronal functions including neuron projection, neural tube development, and axon guidance (Fig 5C, **bottom panel**). Notably, we identify several pluripotent-related genes such as *Eras*, *Lin28a*, and *Utf1* [27–30] in the downregulated differentially expressed genes by GSK-J4. To confirm the regulation of these genes by Utx, we measured the RNA expression of several differentially expressed genes following Utx depletion. Among those genes downregulated by GSK-J4, the expression of *Utf1*, *Eras*, *Zic2*, and *Fgf15* are reduced in Utx depleted cells, whereas, *Zic5* expression does not show a significant change (Fig 6A). Genes that are induced by GSK-J4, *Nodal*, *HoxC13*, and *Cdkn1a*, also show reduced expression following Utx knockdown (Fig 6A). These data suggest that Utx activates the expression of these genes by both demethylase-dependent and-independent mechanisms.

### GSK-J4 inhibits H3K4 demethylation at *Xist*, *Nodal*, and *HoxC13* in female ESCs

Recently, it has been reported that GSK-J4 can inhibit not only Kdm6, but also other JmjC histone demethylase family members [15]. To clarify the effects of GSK-J4, we used qChIP and tested the trimethylation levels of H3K4, H3K9 in addition to H3K27 in female ESCs after GSK-J4 exposure. We observed elevated H3K27me3 levels of all the tested genes except *HoxC13*. No significant changes of H3K9me3 levels were observed after GSK-J4 (Fig 6B). In contrast, only the genes induced by GSK-J4 (*Xist*, *Nodal*, and *HoxC13*) show enhanced H3K4me3 levels, indicating that the demethylation of H3K4me3 of these genes is also inhibited by GSK-J4 accounting for the elevated expression of these genes. Taken together, our high-through put analysis defines the global gene expression changes in female ESCs following GSK-J4 by inhibiting both H3K4 and H3K27 demethylation.

### JmjC histone demethylase activity is a determinant of XCI timing

**Fig 7:**
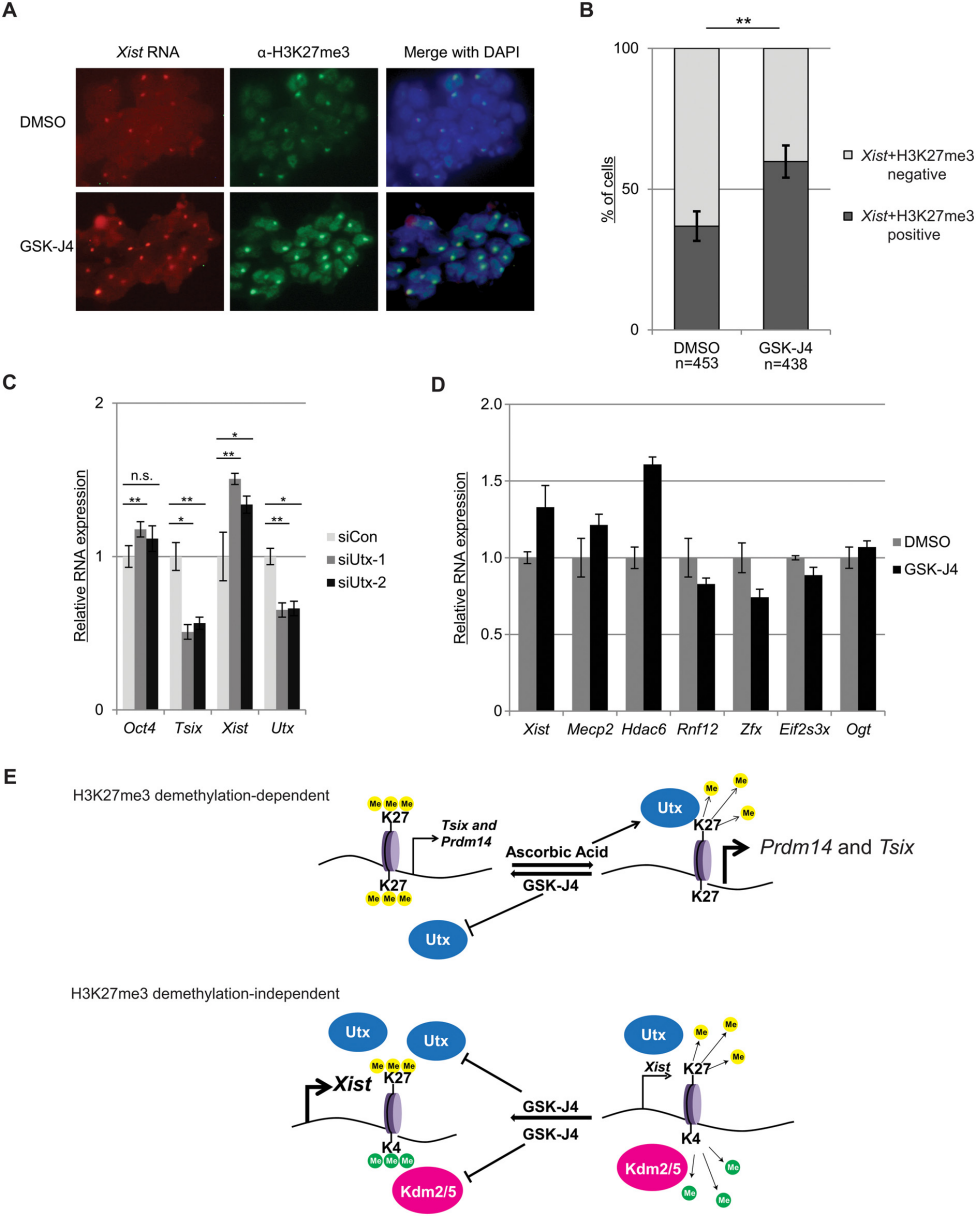
JmjC histone demethylases influence the timing of XCI in female ESCs. (A) *Xist* RNA FISH combined with anti-H3K27me3 immunofluorescence. Female ESCs were cultured in the presence or absence of 10 μM GSK-J4 for 3 hr and then subjected to the induction of differentiation to day 4 EBs as described above. (B) Quantification of the *Xist*/H3K27me3 foci positive cells. The graph represents the percentage of *Xist* + H3K27me3 dual-stained, positive foci (*Xist*+H3K27me3 positive) cells versus the dual-stained negative cells (*Xist*+H3K27me3 negative). The standard deviations are represented as error bars from three independent experiments. The Fisher’s exact test was used for the statistical analysis. “n” indicates the total number of counted cells. **p<0.01. (C) Depletion of Utx induces *Xist* expression in differentiating female ESCs. Female ESCs were transfected with two independent Utx siRNAs and plated onto undifferentiated cellular conditions. Then, the media was changed to N2B27 and a second siRNA transfection was performed 24 hr later. The cells were cultured an additional 24 hr, harvested, and subjected to RT-qPCR. The graph represents the means from three independent experiments with the standard deviations as error bars. The Student’s *t*-test (two tailed unpaired) was used for the statistical analysis. *p<0.05; **p<0.01; n.s. = not significant (p>0.05). (D) The expression of X-linked genes upon GSK-J4 treatment in female MEFs. Female MEFs were treated with GSK-J4 for 24 hr, harvested, and subjected to RT-qPCR. The graph represents the means from three independent experiments with the standard deviations shown as error bars. (E) Working models for the Utx H3K27 demethylase. Utx maintains the expression of a subset of genes such as *Prmd14* and *Tsix* by demethylating H3K27me3. These genes are repressed by GSK-J4 (Upper panel). Ascorbic acid can enhance the demethylase activity of Utx, activating *Prdm14* and *Tsix* (Upper panel) GSK-J4 can also inhibit H3K4me3 demethylases (such as Kdm2 and Kdm5) to induce gene expression of genes such as *Xist* (Lower panel). Utx enriches its target by GSK-J4 via unknown mechanisms, which may activate the demethylase-independent functions of Utx.

We next asked whether histone demethylation is critical for the establishment as well as the maintenance of XCI. To address these questions, we tested whether the inhibition of the JmjC histone demethylases can alter the timing of XCI. Female ESCs were culture in the absence or presence of GSK-J4 for 3 hr and then subjected to differentiation by forming EBs concomitant with LIF removal. At day 4 of differentiation, we evaluated the XCI status using *Xist* RNA fluorescent *in situ* hybridization (RNA FISH) coupled with anti-H3K27me3 immunostaining, marks that decorate the entire female inactive X-chromosome. We then evaluated individual cells for the presence of both *Xist* and H3K27me3 foci (Fig 7A). We found that EBs derived from GSK-J4 treated female ESCs show a statistically higher ratio of both *Xist* and H3K27me3 positive foci (Fig 7B), indicating that the JmjC histone demethylases play an important role in XCI timing.

Next, we clarified the role of Utx in XCI by first depleting Utx by siRNA and then inducing these ESCs to differentiate using N2B27 serum-free media. The expression levels of *Oct4*, *Tsix*, and *Xist* were measured 1 day after the induction of cellular differentiation. Our results show that the knockdown of Utx results in a significant upregulation of *Xist*, whereas the expression of *Tsix* is reduced (Fig 7C). These findings indicate that Utx is a negative regulator of XCI during female ESC differentiation. The expression of *Oct4* did not show an alteration after knockdown of Utx.

Finally, to test the significance of the JmjC histone demethylases for XCI maintenance, we measured the expression of several X-linked genes (*Mecp2*, *Hdac6*, *Rnf12*, *Zfx*, *Eif2s3x*, and *Ogt*) in GSK-J4 treated female MEFs. We observed an increased expression of *Hdac6* after GSK-J4 exposure (Fig 7D). In contrast, the other X-linked genes did not alter expression (Fig 7D). Previous studies have shown that ectopic *Xist* expression induces secondary XCI in *cis*, suggesting that the inhibition of JmjC histone demethylation activates *Xist* from the Xi. Collectively, these results show that the JmjC histone demethylases influence the timing of XCI in female differentiating ESCs but are dispensable for the maintenance of XCI along the X-chromosome.

## Discussion

The dynamics of XCI/XCR in female ESCs provides an excellent model for epigenetic reprogramming between the pluripotent and differentiated states. XCI occurs during cellular differentiation, while XCR transpires during dedifferentiation towards pluripotency. A previous report has linked the *Prdm14* and *Tsix* to XCR but the underlying mechanism for their action is unknown. In this study, we have revealed the pivotal roles of Utx for the expression of *Prdm14*, *Tsix*, and *Xist* in female ESCs. Our qChIP and knockdown studies show that Utx selectively occupies the TSSs of *Prdm14* and *Tsix*, and *Xist* intron 1 and maintains the expression of these genes. Consistently, AA enhances demethylation of H3K27me3 and activates the expression of *Prdm14* and *Tsix* in the absence of GSK-J4, indicating that AA-induced up-regulation of these genes is H3K27 demethylation-dependent. In addition to these genes, we identified genes down-regulated by GSK-J4 using RNA-Seq, including *Utf1*, which has been previously reported as an Utx target [14]. We also found that GSK-J4 exposure increases the expression and H3K4me3 levels of *Xist*, *Nodal*, and *HoxC13*. These results are consistent with the recent report from the Helin lab indicating that GSK-J4 can inhibit both the H3K27 and H3K4 demethylases in cell culture [15]. However, depletion of Utx results in reduced expression of *Xist*, *Nodal*, and *HoxC13*, suggesting that Utx activates these genes in an H3K27me3-independent manner. Taken together, our results show that GSK-J4 alters the gene expression by interfering with both H3K27me3 and H3K4me3 demethylation.

Two different JmjC family members, Kdm2 and Kdm5, demethylate H3K4me3. The Kdm2 family consists of Kdm2a and Kdm2b (also known as Fbxl10) and can also demethylate H3K36me2. Previous studies reveal that Kdm2b binds to GC-rich promoters across the entire genome in male ESCs and regulates PRC1 complex recruitment. The Kdm5 family includes Kdm5a, Kdm5b, Kdm5c, and Kdm5d and is specific for the demethylation of H3K4 [31–34]. We investigated the publically available ChIP-Seq database for Kdm2a, Kdm2b, and Kdm5c [35, 36] binding to see whether their occupancy correlates with the altered gene expression we observed following GSK-J4 exposure. Indeed, 16 of 160 up-regulated genes and only 1 of 29 down-regulated genes following GSK-J4 exposure have significant peaks of Kdm5c. We did not observe a correlation between gene expression alteration and the occupancy of Kdm2a or Kdm2b (data not shown). This may reflect the possibility that GSK-J4 mainly contributes to the inhibition of the Kdm5 rather than Kdm2 family members.

The demethylase-independent role(s) of Utx remain to be elucidated. Notably, Utx constitutes a large complex that harbors components of transcriptional elongation and the chromatin remodeling factors such as Brg1-containing ATPase-dependent Swi/Snf members. [37–39]. Both Utx and Jmjd3 function in demethylase-independent roles for T-box family gene expression [38]. A recent study from the Magnuson lab provides further evidence for a H3K27 demethylase-independent function in the early mouse embryo [40]. Male embryos devoid of KDM6 H3K27 demethylation (*Utx* ^-/y^;*Jmjd3* ^-/-^) survive to term, whereas female embryos (*Utx* ^-/-^;*Jmjd3* ^-/-^) have a developmental delay and exhibit a mid-gestational lethality [40]. It has been proposed that H3K27me3 may be replaced by passive mechanisms such as replication-dependent histone turnover [41–46]. We propose an auto-regulatory mechanism for Utx activity and that the demethylase activity of Utx controls the demethylase-independent activity (Fig 7C).

Our studies reveal that the JmjC histone demethylases influence the timing of XCI in differentiating female ESCs. In contrast to undifferentiated ESCs, the knockdown of Utx followed by the induction of differentiation, results in the upregulation of *Xist*, indicating that Utx functions as a negative regulator of XCI during the cellular differentiation of female ESCs. This may be due to the ability of Utx in maintaining the expression of XCI repressors such as *Tsix* and *Prdm14*. Our findings suggest that the JmjC histone demethylases are dispensable for the maintenance of XCI as the expression of X-linked genes, with the exception of *Hdac6*, are not affected by GSK-J4 treatment in female MEFs. Interestingly, GSK-J4 fails to induce *Xist* expression in male MEFs. This difference may reflect other mechanisms for *Xist* repression in differentiated male cells and the plasticity of the chromatin in ESCs. Collectively, our study provides novel insight of the regulatory mechanisms for the maintenance of pluripotent genes, and preventing XCI by demethylation of H3K27me3 as well as H3K4me3.

## Materials and Methods

### Cell culture

Female LF2 [47] (a gift from Dr. J. Wysocka), EL16.1 ESCs [48]; and male R1 [49] (a gift from Dr. A.K. Hadjantonakis) and J1 ESCs [48], were maintained on mouse embryonic fibroblasts feeders as described [19]. To remove feeders, ESCs were passaged at least once without feeders before experiments. GSK-J4 and L-ascorbic acid (AA) were purchased from R & D Systems and Cayman, respectively. The cells were treated with 10 μM GSK-J4 and/or 50 μg/ml L-ascorbic acid (AA). For the serum-free differentiation of the ESCs, N2B27 media was used as described previously [50].

### Transcriptomic analysis

Female ESCs were exposed to 10 μM GSK-J4 plus or minus 50 μg/ml L-ascorbic acid (AA) for 24 hr. The total RNA was prepared with TRIzol (Life Technologies), treated with DNase I (New England Biolabs), and column-purified (Qiagen). Complementary DNA libraries were prepared and the RNA-sequencing was performed using the Illumina HISeq2500/1500 high-throughput sequencing platform at the Weill Cornell Medical College Genomics Resources Core Facility. Single-end reads were subjected to the removal of duplicates and TopHat2, Cufflinks2, CummeRbunde, and Genome Explore were used through Maser3 to analyze the reads [51–54]. Gene Ontogeny analyses were performed using DAVID [55,56]. The Gene Expression Omnibus (GEO) accession number for the transcriptome analysis is GSE67674.

## Supporting Information

S1 Table List of PCR primers. (PDF)

S1 Fig Jmjd3 protein expression is not affected by proteasome inhibitor in ESCs. Embryonic stem cells (ESCs) were treated with 50 mM MG-132 for 3 hr and subjected to western blot with anti-Jmjd3 antibodies. Anti-c-Myc and anti-pan-H3 antibodies were used as positive and loading controls, respectively.
(PDF)

S2 Fig Alteration of gene expression by GSK-J4 treatment in two independent male and female ESC lines. J1 (male) and EL16.7 (female) mouse ESCs were treated with GSK-J4 and subjected to RT-qPCR. The graphs are shown as the mean values of three independent experiments. Error bars represent one standard deviation.
(PDF)

S3 Fig *Xist* expression is not induced by GSK-J4 treatment in male MEFs. Male MEFs were treated with 10 μM GSK-J4 for 24 hr, harvested, and subjected to RT-qPCR. The mean values of three independent experiments were represented with standard deviations shown as error bars.
(PDF)

S4 Fig Utx protein amount is not increased following GSK-J4 or L-ascorbic acid (AA) treatment. (A) Female ESCS were treated with GSK-J4 and then subjected to western blot using anti-Utx and pan-histone H3 antibodies. (B) Female ESCs were treated with AA and then subjected to western blot using anti-Utx and anti-Actin antibodies.
(PDF)

S5 Fig RNA-Seq validation. (A) Expression of *Oct4*, *Nanog*, *Prdm14*, and *Tcl1* are shown as FKPM (Fragments per Kilobase of exon PFont>Symboler Million mapped fragments (B) Mapping of RNA read fragments at *Xist* in DMSO- versus GSK-J4-treated female ESCs.).
(PDF)
