# Supplementary material for: Histone Demethylation Maintains Prdm14 and Tsix Expression and Represses Xist in Embryonic Stem Cells
Source: PLoS One. 2015 May 20;10(5):e0125626. doi: 10.1371/journal.pone.0125626 (PMC4439117; doi:10.1371/journal.pone.0125626)
Supplement: S1 Table — (PDF) [file pone.0125626.s001.pdf]

Table S1. List of PCR primers

primers	sequence
RT-qPCR	
UtxFwd	GACAGCGGAGGAGAGGGAG
UtxRev	CGAACAGCCTTGCCCAGC
Jmjd3Fwd	CTCTGGAACTTTCATGCCGG
Jmjd3Rev	CTTAGCCCCATAGTTCCGTTTG
Oct4Fwd	CACGAGTGGAAAGCAACTCA
Oct4Rev	AGATGGTGGTCTGGCTGAAC
NanogFwd	ACCTGAGCTATAAGCAGGTTAAGAC
NanogRev	GTGCTGAGCCCTTCTGAATCAGAC
Prdm14Fwd	GCATCCTGGTTCCCACAGAG
Prdm14Rev	CTGCAGAACACGCCAAAGTG
Tcl1Fwd	CAGTGGTGTCTGTGCCTCTG
Tcl1Rev	AGGTCTTGTCCCTCATGGTG
TsixFwd	GGTAACAATTTTCCCGCCATGTG
TsixRev	GGAAATAAACGGAACGCAGTACC
XistFwd	CAGAGTAGCGAGGACTTGAAGAG
XistRev	CCCGCTGCTGAGTGTTTGATATG
18SrRNAFwd	GTAACCCGTTGAACCCCATT
18SrRNARev	CCATCCAATCGGTAGTAGCG
ChIP-qPCR	
Oct4TSSFwd	TGGGCTGAAATACTGGGTTC
Oct4TSSRev	GTCCTTACAGCCCACTCAGC
NanogTSSFwd	GAAGATTTCCCCAGGTTTCC
NanogTSSRev	GACCTTGCTGCCAAAGTCTC
Prdm14TSSFwd	CAGGGAACGGTCAAGAGAAC
Prdm14TSSRev	CTGTGGAGGTCCCCTGATAG
Tcl1TSSFwd	CGAGCACAAAGACAGCAAAC
Tcl1TSSRev	TCTGTCCTTTTTGGGTGTCC
TsixTSSFwd	ACCCAGTCTTTGAGACCGTAAG
TsixTSSRev	AGCTCCAACACAGTCTCATTTG
XistTSSFwd	AGATGCCAATGACCCAAAAC
XistTSSRev	CGACCTCAGATGAGGAGAGG
Xist-int1Fwd	CTGAAGATGGTGATGGCGAGTTG
Xist-int1Rev	AAAGAGTTCCCCAAATTAGTGTCCTG
