# Supplementary figures and images for: Histone Demethylation Maintains Prdm14 and Tsix Expression and Represses Xist in Embryonic Stem Cells
Source: PLoS One. 2015 May 20;10(5):e0125626. doi: 10.1371/journal.pone.0125626 (PMC4439117; doi:10.1371/journal.pone.0125626)

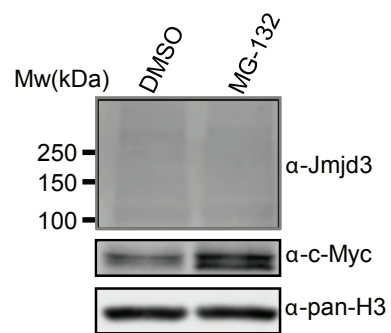

Supplement: S1 Fig — Embryonic stem cells (ESCs) were treated with 50 mM MG-132 for 3 hr and subjected to western blot with anti-Jmjd3 antibodies. Anti-c-Myc and anti-pan-H3 antibodies were used as positive and loading controls, respectively. (PDF) [file pone.0125626.s002.pdf]

**A**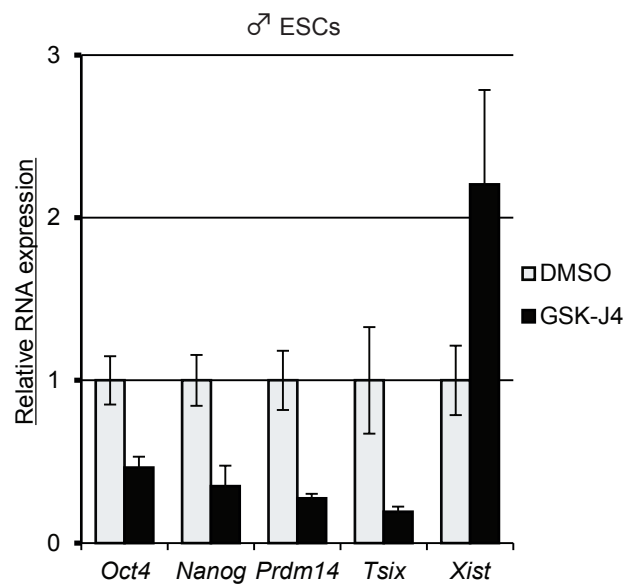**B**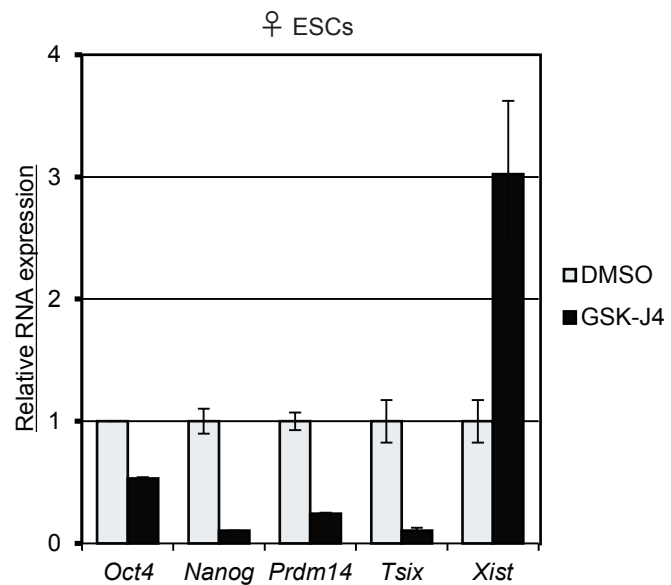

Supplement: S2 Fig — J1 (male) and EL16.7 (female) mouse ESCs were treated with GSK-J4 and subjected to RT-qPCR. The graphs are shown as the mean values of three independent experiments. Error bars represent one standard deviation. (PDF) [file pone.0125626.s003.pdf]

Male MEFs

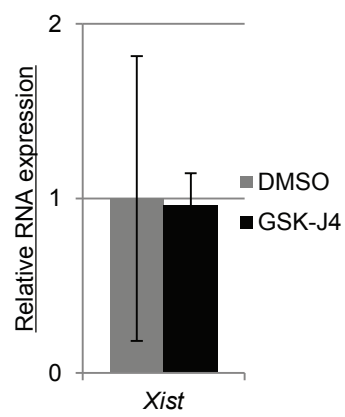

Supplement: S3 Fig — Male MEFs were treated with 10 μM GSK-J4 for 24 hr, harvested, and subjected to RT-qPCR. The mean values of three independent experiments were represented with standard deviations shown as error bars. (PDF) [file pone.0125626.s004.pdf]

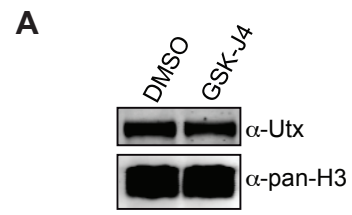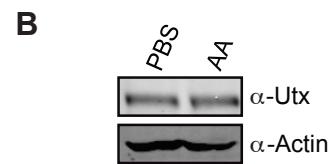

Supplement: S4 Fig — (A) Female ESCS were treated with GSK-J4 and then subjected to western blot using anti-Utx and pan-histone H3 antibodies. (B) Female ESCs were treated with AA and then subjected to western blot using anti-Utx and anti-Actin antibodies. (PDF) [file pone.0125626.s005.pdf]

**A**

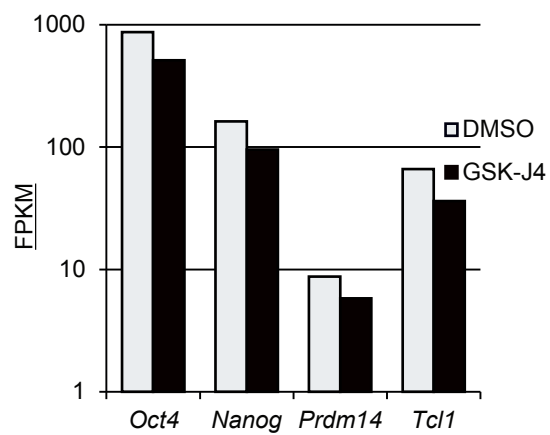

**B**

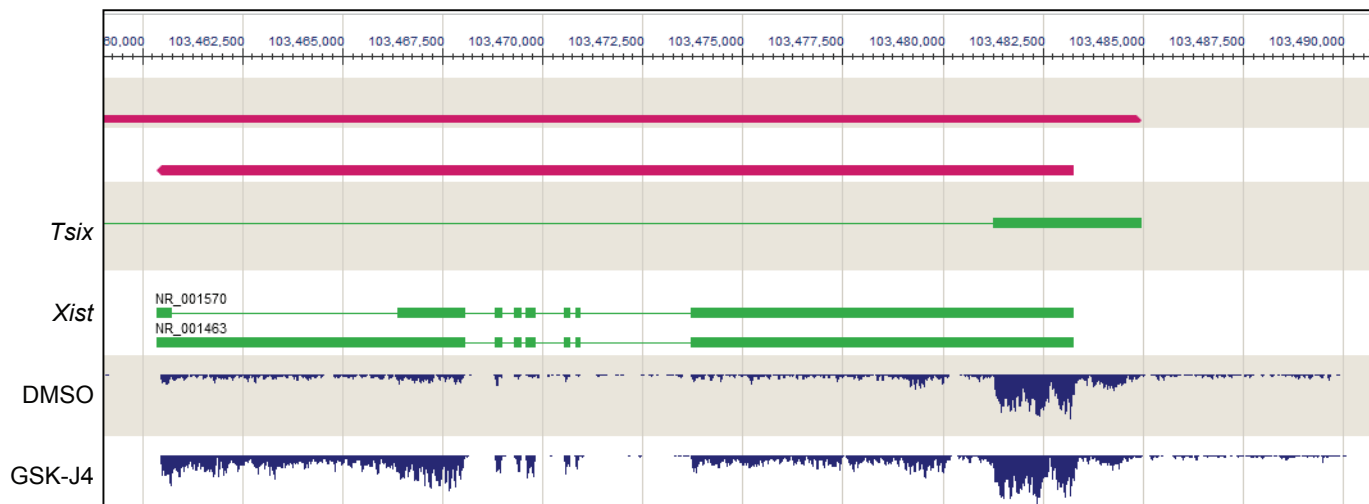

Supplement: S5 Fig — (A) Expression of Oct4, Nanog, Prdm14, and Tcl1 are shown as FKPM (Fragments per Kilobase of exon PFont>Symboler Million mapped fragments (B) Mapping of RNA read fragments at Xist in DMSO- versus GSK-J4-treated female ESCs.). (PDF) [file pone.0125626.s006.pdf]
